# Shexiang tongxin dropping pill alleviates myocardial injury induced by coronary microembolization by down-regulating APOC1 to inhibit STAT3 signaling pathway

**DOI:** 10.18632/aging.205796

**Published:** 2024-05-20

**Authors:** Yangui Wang, Tao Wang, Tingting Ma, Jin Zhao, Weigang Qi

**Affiliations:** 1 Department of General Practice, Shanghai Pudong New Area People’s Hospital, Pudong New Area 201299, Shanghai, P.R. China

**Keywords:** shexiang tongxin dropping pill, APOC1, STAT3 signal pathway, CME, myocardial injury

## Abstract

Aim: This study determines to validate the mechanism of Shexiang Tongxin dropping pill (STDP) in attenuating coronary microembolization (CME) induced myocardial injury.

Methods: CME rat models were established and underwent corresponding treating. Gene chip analysis was performed in rat myocardial tissues for GO and KEGG enrichment analysis. The differentially expressed genes were detected by qRT-PCR. H&E staining and ELISA were used for pathological analysis and detection of troponin (cTnI) and Creatine Kinase Isoenzyme (CK-MB). Lipopolysaccharide (LPS) treated primary cardiomyocytes were used to mimic inflammatory *in vitro* models. Cell viability and apoptosis of cardiomyocytes were determined by MTT and flow cytometry. The expressions of inflammatory cytokines, apoptotic proteins and proteins related to the STAT3 signal pathway were detected by western blot. APOC1 mRNA expression was detected by qRT-PCR. Immunofluorescence (IF) was used for subcellular localization of p-STAT3 and the binding of APOC1 with STAT3 was verified using Co-IP.

Results: STDP can attenuate myocardial injury in CME rat models, and lead to decreased expression of APOC1 and suppressed STAT3 signal pathway. *In vitro* models found STDP can suppress the cell viability and cell apoptosis of primary cardiomyocytes, in addition to suppressing the secretions of IL-6, IL-1β and TNF-α, while the protective effect of STDP can be reversed by overexpression of APOC1. Co-IP found that APOC1 can bind STAT3 directly. APOC1 can increase p-STAT3 expression in the nucleus to activate the STAT3 signal pathway.

Conclusions: STDP can suppress APOC1 and STAT3 signal pathway to inhibit inflammation and cell apoptosis of cardiomyocytes. APOC1 may be one of the key regulatory factors in CME-induced myocardial injury.

## INTRODUCTION

Obstruction and dysfunction of the coronary microcirculation can lead to patchy microinfarcts accompanied by inflammation, contributing to progressive impairment of myocardial contractile function [1]. Microemboli-induced coronary microcircular obstruction is commonly identified along with epicardial coronary atherosclerotic plaque rupture or fissuring [2]. Coronary microembolization (CME) has been recognized as an iatrogenic complication following percutaneous coronary interventions (PCIs) that occurs along with a detachment of atherosclerotic plaque debris [3]. Also, CME leads to microvascular obstruction post-reperfused acute myocardial infarction, involving physical obstruction, vascular contraction, and inflammatory response in the coronary microcirculation [1]. CME following PCI can result in post-procedural microinfarcts, increased cardiac biomarkers, and impaired cardiac function [4]. CME induces infiltration of numerous inflammatory cells into the myocardium as well as the secretion of multiple inflammatory factors, which consequently results in local inflammation in the myocardium; these consequences represent the main causes of myocardial injury and main progressive cardiac adverse events [5].

Recently, traditional Chinese medicine (TCM) has attracted great attention owing to its apparent advantages in improving cardiac function and quality of life [6, 7]. Although randomized controlled trials are currently deficient to offer a reliable scientific basis for its application in disease prevention and treatment, TCM still shows great potential as a complementary and alternative strategy for cardiovascular disorders [8]. For instance, NaoXinTong Capsule (NXT) has been regarded as a cardioprotective prescription for cardiovascular diseases, including CME, and its performance is linked to its effect on inflammation, apoptosis, oxidative stress, angiogenesis, insulin resistance, lipid/glucose metabolism, etc. [9].

Shexiang Tongxin dropping pill (STDP), an extensively used TCM preparation for coronary heart disease (CHD), has shown great cardioprotective beneficial in cardiovascular diseases, including chronic heart failure [10], coronary microvascular dysfunction [11], and myocardial ischemic injury [12]. Of note, an existing study has demonstrated that STDP can attenuate CME via mediating mitochondrial permeability transition pore opening and dephosphorylating AKT-GSK3β [13]. This study established a rat model of CME for mechanistic investigations. Given the broad application of high-throughput sequencing in the identification of genomics, epigenomics, and transcriptomics in cardiovascular diseases [14], we employed this method to screen the STDP-mediated genes in CME rat models, to determine the possible molecular mechanisms underpinning the cardioprotective function of STDP. Additionally, an *in vitro* model was induced by lipopolysaccharide (LPS) for further validation of the cardioprotective mechanisms.

## MATERIALS AND METHODS

### CME rat models [15]

Specific pathogen-free (SPF) male SD rats (n = 30, 370 ~ 400 g) were purchased from Hunan SJA laboratory Animal Co., Ltd and randomly classified into normal control group (sham group), CME model group (Model group) and CME + STDP group (Model+STDP group).

Rats in Model+STDP group were gavaged with STDP (40 mg/kg/d) one week before modeling. Rats in Model and Model+STDP groups were anesthetized with 2% pentobarbital sodium (0.2 mL 100g) through intraperitoneal injection. Rats underwent endotracheal intubation for assisted ventilation after skin disinfection and fur shaving. The chest was opened to expose the root of the aorta. The ascending aorta was blocked for 30 s, during which the left ventricle was injected with 1.0 mg/kg sodium laurate (Fluka) using a 29 probe and the ascending aorta was clamped for 10 s before the chest was closed. The rats in the sham group were injected with normal saline. After 1.5 h, the thrombosis was detected. After modeling for 24 h, rats were anesthetized with their blood in the abdominal aorta collected for centrifugation (3000 r/min, 4° C) for 15 min. The serum collected was used for biochemical analysis and ELISA. Then the rats were sacrificed and the hearts were collected for further analysis.

### High-throughput sequencing (NGS)

The myocardial tissues from rats in the sham group, Model group and Model+STDP group were collected for RNA extraction. Specifically, the tissues were homogenized in a 1600 MiniG®-Automated Tissue Homogenizer (Metuchen, NJ, USA) with TRIzol® reagent (Thermo Fisher Scientific Inc., Waltham, MA, USA). The quality of RNA extracted was tested using a NanoDrop™ 2000 spectrophotometer (Thermo Fisher Scientific). Total RNA (1 μg) was used as the input for mRNA synthesis. mRNAs were purified using magnetic beads bound oligo-dTs, and fragmented into 180 ~ 250 nt. RNAs were used as the templates to synthesize the first-strand cDNA. A DNA library was constructed on the Illumina NovaSeq 600 platform (Illumina, San Diego, CA, USA) through end-repair, adaptor-ligation, and PCR amplification.

### Cardiac function detection [16, 17]

After the last STDP treatment, the rats were fasted for 12 h and then underwent cardiac function detection using a Philips sonos7500 ultrasonic apparatus (Philips Technologies, Amsterdam, NY, USA). The rats were intraperitoneally injected with 2% pentobarbital sodium (0.2 mL/100g) and after about 10 to 15 min, the rats were successfully anesthetized. The probe was placed on the antetheca of the rat’s left chest with a frequency of 10 MHz. The views of the left ventricular long axis, apical four chambers and two chambers were selected to detect the left ventricle end-systolic volume (ESV), left ventricular end-systolic dimension (LVIDs), interventricular septal (IVS), end-diastolic volume (EDV), left ventricular internal diastolic dimension (LVIDd), left ventricular end-diastolic diameter (LVEDd), left ventricular posterior wall thickness (LVPW) and cardiac output (CO). The calculations were performed for three cardiac cycles and averaged. Left ventricular ejection fraction (LVEF) = (EDV-ESV)/EDV*100% and left ventricular fractional shortening (LVFS) = (LVIDd-LVIDs)/LVEDd*100%. All detections were performed by an experienced doctor in the ultrasonic department who is blinded to the experimental design.

### CK-MB and cTnI detection [15, 17]

The blood collected from the abdominal aorta was centrifuged (3000 r/min, 15 min). Expression of CK-MB in the serum was determined using an automatic biochemistry analyzer (Cobas8000, Roche, USA). The expression of cTnI in rats was detected using a Cardiac Troponin I ELISA kit (ab246529, Abcam, UK).

### H&E staining [15, 17]

H&E staining kit (C0105M, Beyotime, China) was used. The myocardial tissues were fixed in 10% formalin and embedded in paraffin before slicing. The slices were dewaxed by xylene and then treated with graded ethyl alcohol to water, followed by washing in running water. After that, the slices were stained in hematoxylin solution for 5 ~ 10 min and then washed in distilled water to remove the excessive staining solution. Then 0.5 ~ 1% hydrochloric acid alcohol was used for color separation till the nuclear and chromatin can be visualized under a microscope. Slices were washed in running water and re-stained in 0.5% eosin staining for 2 min, followed by dehydration under 70%, 80%, 90% and 100% ethyl alcohol, and transparentized in xylene twice, each for 5 min. Neutral gum or other mounting medium was used before the slices were observed under a microscope (Zeiss, Germany). The nuclear was blue, while the cytoplasm was pink or red.

### Separation and culture of rat primary cardiomyocytes [5]

SD rats (1 ~ 2 days, purchased from the Animal Center of Xiangya Hospital) were dissected to obtain their hearts placed in cold PBS. Then the heart tissues were digested in collagenase II (Sigma-Aldrich St. Louis, MO, USA) and trypsin for 5 min and the supernatant was collected into a 15 mL centrifuge tube in which 8 mL complete culture medium was prepared. The digestion process was repeated until tissues were fully dissolved. The supernatant was placed in one tube for centrifugation (1000 rpm, 8 min) to remove the supernatant. The cells were then re-suspended at 37° C for 1.5 h using differential adhesion method to remove the fibroblasts. The remaining cells were cultured in DMEM (Gibco, USA), in which 10% fetal bovine serum (FBS, Gibco) and 1% penicillin-streptomycin (Gibco) were prepared. The culture medium was refreshed every 3 days. The primary cardiomyocytes were stained by cTnI (ab47003, Abcam) with the standards of 90% positive rate as successfully isolated. The primary cardiomyocytes were cultured in an incubator at 37° C with 5% CO_2_. Lipofectamine 2000 reagent (Invitrogen, Carlsbad, CA, USA) was used for cell transfection. After cell transfection for 48 h, cells were treated with or without 10 μg/mL LPS treatment for 12 h.

si-NC (5’-GATGAAGAGCACCAACTC-3’), si-APOC1-1 (5’-GCGCAAUGGAGAGCUUACCGGAUAA-3’) and si-APOC1-2 (5’-GGCAGCCAUUGAGCAUAUCAAACA-3’) were synthesized by Sangon Biotech (Shanghai, China). pCMV-Flag-APOC1 and pCMV-Flag-STAT3 were synthesized by GenePharma (China).

### Reverse transcription quantitative polymerase chain reaction (qRT-PCR)

The total RNA in tissues and cells was isolated using TRIZOL (Invitrogen, Carlsbad, CA, USA) and subjected to reserve transcription using RT kit (TaKaRa, Tokyo, Japan) based on the instruction. The relative expression of target genes was detected using a LightCycler 480 PCR instrument (Roche, Indianapolis, IN, USA) under the conditions instructed in the PCR kit (SYBR Green Mix, Roche Diagnostics, Indianapolis, IN, USA): 95° C for 10s, 45 cycles of 95° C for 5s, 60° C for 10s and 72° C for 10s, 72° C extension for 5 min. Three repetitions were set for each PCR reaction and GAPDH was used as the internal control. Data were analyzed using 2^-ΔΔCt^ method: ΔΔCt = experimental group (Ct target gene –Ct internal control) – control group (Ct target gene-Ct internal control). The primer sequences for genes are listed in Table 1.

**Table 1:** Primer sequences for reverse transcription quantitative polymerase chain reaction.

**Name of primer**	**Sequences (5’-3’)**
APOC1-F	CTAGTGATGCCCCCTATCCG
APOC1-R	ATGGCTACGACCACGATCAG
TNF-α-F	AACACACGAGACGCTGAAGT
TNF-α-R	TCCAGTGAGTTCCGAAAGCC
IL-6-F	CATTCTGTCTCGAGCCCACC
IL-6-R	GCAACTGGCTGGAAGTCTCT
GAPDH-F	TGCACCACCAACTGCTTAG
GAPDH-R	GATGCAGGGATGATGTTC
IL-1β-F	GACTTCACCATGGAACCCGT
IL-1β-R	GGAGACTGCCCATTCTCGAC

R, reverse, F, forward.

### Western blot

RIPA lysis from Beyotime (Jiangsu, China) was used for cell lysis and the concentration of protein was detected using a BCA kit (Beyotime). The protein was mixed with loading buffer (Beyotime) in boiling water bath for 3 min for denaturing. The protein then underwent electrophoresis at 80 V for 30 min and then 120 V for 1 ~ 2 h. The membrane transference was performed in an ice water bath at the current of 300 mA for 60 min, followed by washing for 1 ~ 2 min, blocking at room temperature for 60 min or at 4° C overnight. After that, the membranes were incubated with the primary antibodies of APOC1 (ab189866, 1:1000), p-STAT3 (ab76315, 1:1000), STAT3 (ab68153, 1:1000), Cleaved caspase-3 (ab2302, 1:1000), BAX (ab32503, 1:1000), Bcl-2 (ab194583, 1:1000) and GAPDH (ab8245, 1:5000) for 1 h at room temperature at a shaking table. The membranes were washed for 3 × 10 min and then incubated for 1 h at room temperature with goat anti-rabbit secondary antibody IgG (ab6702, 1:5000) or goat anti rat IgG (ab6708, 1:5000) before washing for 3 × 10 min. All primary antibodies were from Abcam. A developing solution was added for color development and the images were detected using a chemiluminescence imaging system (Bio-Rad, Hercules, CA, USA).

### Immunofluorescence (IF) [18]

The cardiomyocytes were washed in PBS and then fixed in 4% paraformaldehyde for 10 min. After that, cells were washed in PBS 3 times and then transparentized in PBS containing 0.5% TritonX-100 for 5 min, followed by PBS washing and BSA blocking at room temperature for 0.5 h. Cells were then incubated with anti p-STAT3 antibody (ab76315, 1:500, Abcam) at 4° C overnight. After being washed in PBS three times, fluorescence-labeled secondary antibody (Proteintech, China) was added for incubation at room temperature for 1 h before DAPI (C1002, Beyotime, China) was added for nuclear staining for 5 min. Cells were then washed in PBS for 3 times and sealed with an anti-fluorescence quenching agent. The images were captured under 5 different fields with an FV-1000/ES confocal microscope. Each experiment was repeated 3 times.

### Co-immunoprecipitation (Co-IP) [18]

Cells in each group were treated with IP lysis (P0013, Beyotime) and centrifuged to collect the supernatant. The cell lysate was then incubated with STAT3 antibody (ab119352, 1:50, Abcam) or p-STAT3 antibody (ab76315, 1:20, Abcam) at 4° C overnight. IgG antibody (ab205718, 1:50, Abcam) was used as the negative control. The cell lysate was added to protein A/G beads for incubation at 4° C for 1 h and the pre-cold IP lysis was used to wash the beads. The protein expression of APOC1 was detected using western blot.

### MTT assay

The MTT Assay Kit (Beyotime, Jiangsu, China) was used for cell viability measurement. Cells in each group were seeded in 96 well plates in which 100 μL diluted cell suspension (1×10^5^ cells/mL) was seeded in advance. Three duplicates were set for each sample. After cells were incubated in an incubator for 0 h, 24 h, 48 h, 72 h and 96 h, 20 μL MTT reagent was added to each well at each time point for cell culture of 4 h. After the supernatant was removed, cells were reacted with DMSO (150 μL per well) before the absorbance at 490 nm was measured using Bio-Rad 550 microplate reader (Bio-Rad, Hercules, CA, USA).

### Cell apoptosis detection

AnnexinV-FITC/PI staining was used to detect cell apoptosis. The primary cardiomyocytes were seeded in 6-well plates at a density of 2 × 10^5^ cells per well. Cells were transfected into blank, negative control and transfection groups. About 48 h after cell transfection, the supernatant was removed and cells were washed with 4° C pre-cold PBS before trypsinization. Then cells were collected for centrifuge at 800 g with the supernatant being removed. After PBS washing for 2 times, cells were re-suspended in 500 μL binding buffer based on the instruction of the cell apoptosis kit (AnnexinV-FITC Apoptosis Detection Kit I, BD, USA) and mixed with 5 μL FITC and 5 μL PI without light exposure for 15 min. The cell apoptosis rate was calculated using flow cytometry (BD FACSCalibur).

### Statistical analysis

Each experiment was repeated 3 times and duplicate wells were set for each experiment. Data analysis was performed using GraphPad Prism 6 and data were expressed as mean ± standard deviation. The analysis between two groups was performed using dependent sample *t* test while comparison among multiple groups was analyzed using one way-ANOVA. P-value of less than 0.05 considered as significant differences.

## RESULTS

### High-throughput sequencing

**Figure 1:**
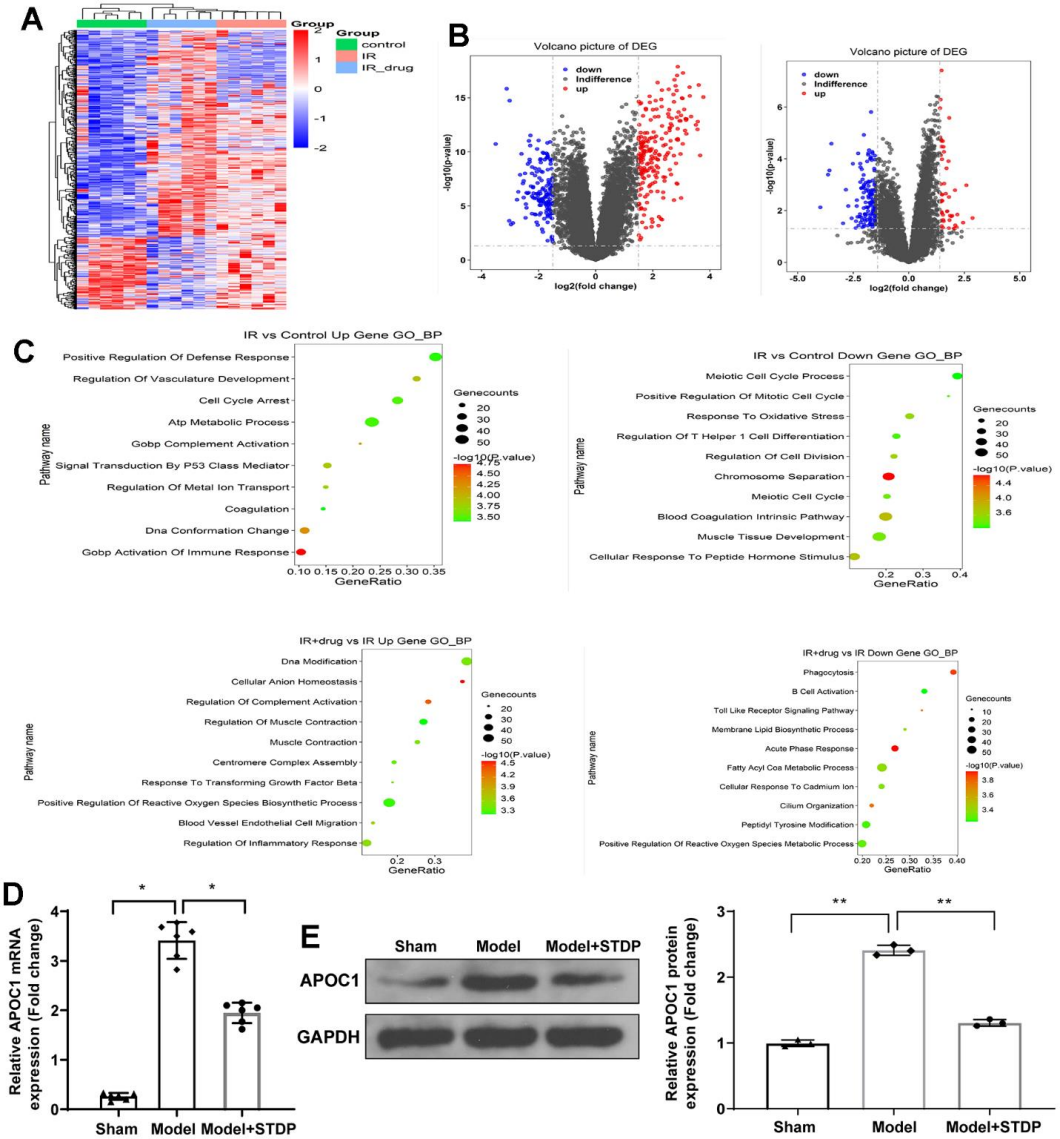
**STDP regulates APOC1 expression in the myocardial tissues of CME rat models.** Note: (**A**) Gene expression levels are shown in a heat map, in which each row represents a gene and each column represents a sample of hip joint capsule tissue. The variation in color of red to blue indicates the signal level, with red indicating high signal or upregulation, blue indicating low signal or downregulation. (**B**) The distribution of differential expressed genes is showed by volcano map. (**C**) GO analysis on the differential expressed genes. (**D**) qRT-PCR detected the APOC1 expression in myocardial tissues. (**E**) Western blot detected the APOC1 protein expression in myocardial tissues. *P < 0.05, ** P < 0.01. Each group had 6 rats and each experiment was repeated for 3 times. STDP, Shexiang Tongxin dropping pill; CME, coronary microembolization.

The myocardial tissues from rats were collected from sham, Model and Model+STDP groups. The volcano, heat map and GO analysis are shown in (Figure 1A–1C). Based on the data obtained from high-throughput sequencing, APOC1 expression was increased by 3.41 folds in myocardial tissues of CME rat models, which can be suppressed by STDP to 1.95. qRT-PCR and western blot further validated this result (Figure 1D, 1E). Those results showed that APOC1 may be involved in STDP attenuating CME-induced myocardial injury.

### STDP can attenuate CME-induced myocardial injury in rats and suppress the STAT3 signal pathway

**Figure 2:**
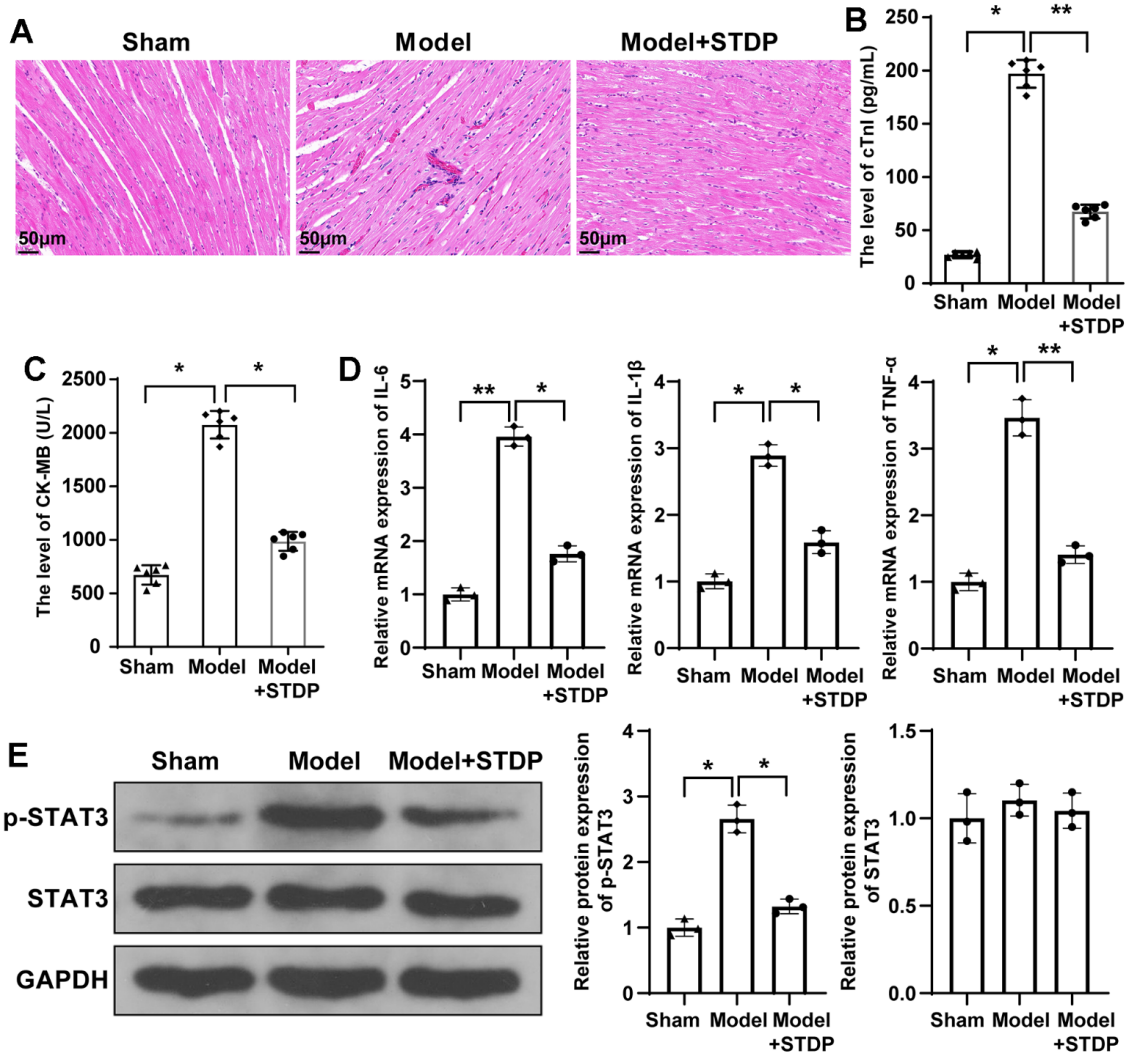
**STDP can attenuate myocardial injury in CME rats and regulate STAT3 signal pathway.** Note: (**A**) H&E staining on myocardial tissues, scale bar of 100 μm; (**B**, **C**) Serum levels of cTnI and CK-MB detected by ELISA; (**D**) qRT-PCR detected the expressions of inflammatory cytokines IL-6, IL-1β and TNF-α in myocardial tissues; (**E**) Western blot detected the protein expressions of STAT3 signal pathway related proteins. *P < 0.05, ** P < 0.01. STDP, Shexiang Tongxin dropping pill; CME, coronary microembolization. cTnI, troponin; CK-MB, Creatine Kinase Isoenzyme.

The myocardial tissues of CME rat models were collected for H&E staining to assess the pathological changes in rats. The staining showed that the myocardial tissues of rats in the Model group were disorderly arranged and surrounded by edema. In addition to that, inflammatory infiltration and erythrocyte diapedesis were also found in the Model group, which can be attenuated by STDP treatment (Figure 2A). The ultrasonic cardiogram showed that the cardiac function of rats in the Model group was suppressed, including left ventricular systolic dysfunction and left ventricular dilation, evidenced by decreased LVEF, LVFS, CO and LVEDD, while the cardiac function of rats in Model+STDP group was improved compared with Model group (Table 2). ELISA detecting the serum levels of cTnI and CK-MB showed that cTnI and CK-MB were increased in the Model group, but suppressed in the Model+STDP group (Figure 2B, 2C). The detection of inflammatory cytokines IL-6, IL-1β and TNF-α by qRT-PCR showed that the Model group had elevated expressions of IL-6, IL-1β and TNF-α, while those expressions in the Model+STDP group were suppressed (Figure 2D). Western blot showed elevated expression of p-STAT3 in Model group, which can be reversed by STDP treatment, but STDP had no significant effect on regulating the expression of STAT3 (Figure 2E). Those results showed that STDP can suppress STAT3 signal pathway to suppress the inflammation in the myocardial tissues of rats.

**Table 2:** Changes in cardiac function (x ± s).

**Group**	**n**	**LVEF (%)**	**LVFS (%)**	**CO (L/min)**	**LVEDd (mm)**
sham	6	81.63 ± 3.57	42.48 ± 4.42	0.189 ± 0.024	5.56 ± 0.39
Model	6	56.64 ± 3.49*	20.18 ± 2.67*	0.101 ± 0.009*	7.91 ± 0.58*
Model+STDP	6	69.87 ± 3.72#	35.13 ± 4.06#	0.164 ± 0.018#	6.68 ± 0.47#

Note: LVEF, left ventricle ejection fraction; CO, cardiac output; LVEDd, left ventricular end-diastolic diameter; LVFS, left ventricle fractional shortening; CME, coronary microembolization. * p < 0.05 compared with the sham group; # p < 0.05 compared with the Model group.

### STDP inhibits LPS-induced inflammation and cell apoptosis in addition to suppressing APOC1 and STAT3 signal pathway

**Figure 3:**
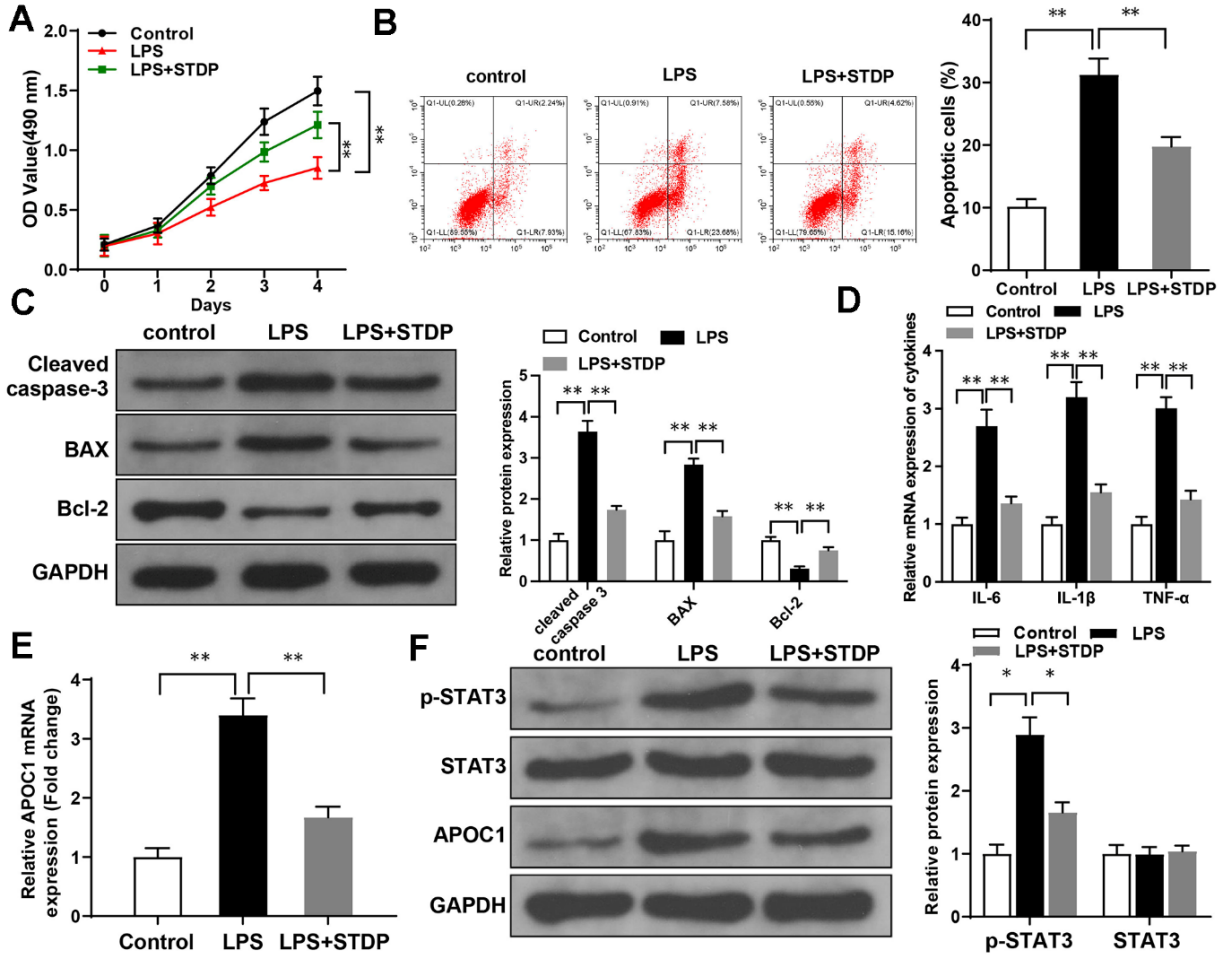
**STDP can reverse LPS-induced cell inflammation and apoptosis in cardiomyocytes.** Note: (**A**) MTT assay detected the viability of rat primary cardiomyocytes; (**B**) AnnexinV/PI staining and flow cytometry analyze the cell apoptosis of cardiomyocytes; (**C**) Western blot detected the expressions of Cleaved caspase-3, BAX and Bcl-2; (**D**, **E**) qRT-PCR detected the expressions of APOC1, IL-6, IL-1β and TNF-α in LPS-induced cardiomyocytes; (**F**) Western blot detected the expressions of STAT3 signal pathway related proteins. *P < 0.05, ** P < 0.01. Each experiment was repeated for 3 times. STDP, Shexiang Tongxin dropping pill; LPS, lipopolysaccharide.

The primary rat cardiomyocytes were treated by LPS to establish *in vitro* cell injury models. MTT assay showed that the viability of cardiomyocytes can be suppressed by LPS treatment, but the cell viability can be rebounded after STDP treatment (Figure 3A). Annexin V/PI staining and flow cytometry showed that LPS can promote cell apoptosis while STDP can attenuate LPS-induced cell apoptosis (Figure 3B). Detection on apoptotic proteins by western blot showed elevated expressions of Cleaved caspase-3 and BAX, and decreased expression of Bcl-2 in LPS treated cells, while such expression profile was reversed in response to STDP treatment (Figure 3C).

qRT-PCR showed the expressions of IL-6, IL-1β, TNF-α and APOC1 can be increased by LPS treatment, but suppressed after STDP treatment (Figure 3D, 3E). Western blot showed that LPS can increase the expressions of p-STAT3 and APOC1 in the cardiomyocytes, but showed no effect on STAT3 expression. The promotive effect of LPS can be suppressed by STDP treatment (Figure 3F). These results showed that STDP can suppress APOC1 and STAT3 signal pathway to attenuate LPS-induced inflammation and apoptosis of cardiomyocytes.

### Overexpression of APOC1 can reverse the protective effect of STDP on cardiomyocytes

**Figure 4:**
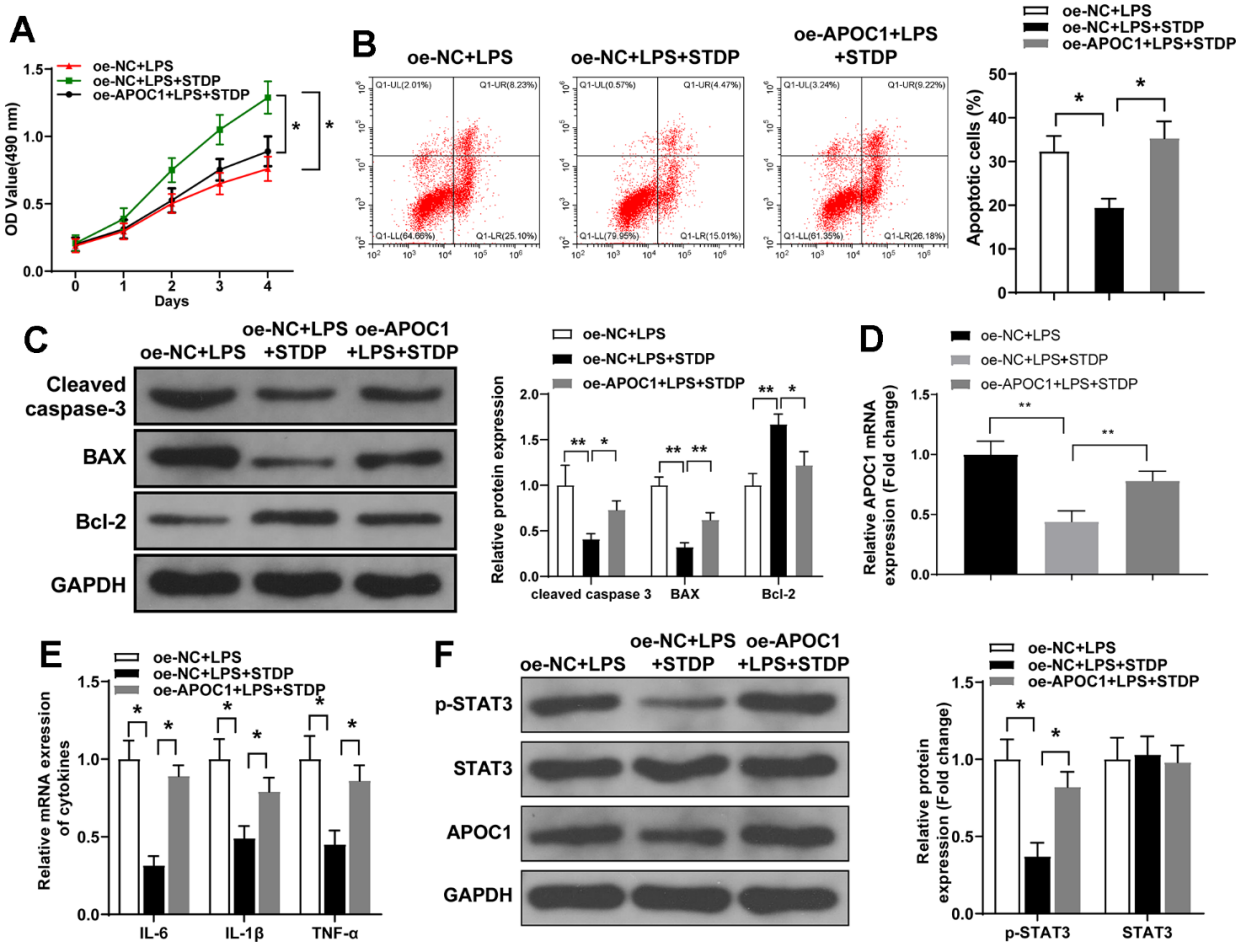
**STDP regulates APOC1 expression to attenuate LPS-induced inflammation and apoptosis in cardiomyocytes.** Note: (**A**) MTT assay detected the viability of rat primary cardiomyocytes; (**B**) AnnexinV/PI staining and flow cytometry analyze the cell apoptosis of cardiomyocytes; (**C**) Western blot detected the expressions of Cleaved caspase-3, BAX and Bcl-2; (**D**, **E**) qRT-PCR detected the expressions of APOC1, IL-6, IL-1β and TNF-α in LPS-induced cardiomyocytes; (**F**) Western blot detected the expressions of STAT3 signal pathway related proteins. *P < 0.05, ** P < 0.01. Each experiment was repeated for 3 times. STDP, Shexiang Tongxin dropping pill; LPS, lipopolysaccharide.

APOC1 overexpression plasmid was transfected into cardiomyocytes and MTT assay showed that STDP can reverse the suppressive effect of LPS-induced cell viability, but this effect can be further abolished by APOC1 overexpression (Figure 4A). Flow cytometry and qRT-PCR demonstrated that STDP can suppress LPS-induced cell apoptosis and inflammation, while overexpression of APOC1 can abolish the effect of STDP on cardiomyocytes (Figure 4B–4E). Western blot demonstrated that STDP treatment can inhibit LPS activated STAT3 signal pathway, which can be further abolished by APOC1 overexpression (Figure 4F). These results showed that APOC1 may be a key regulator underlying the mechanism of STDP attenuating the inflammation and apoptosis in cardiomyocytes.

### APOC1 can bind STAT3 to activate STAT3 signal pathway

**Figure 5:**
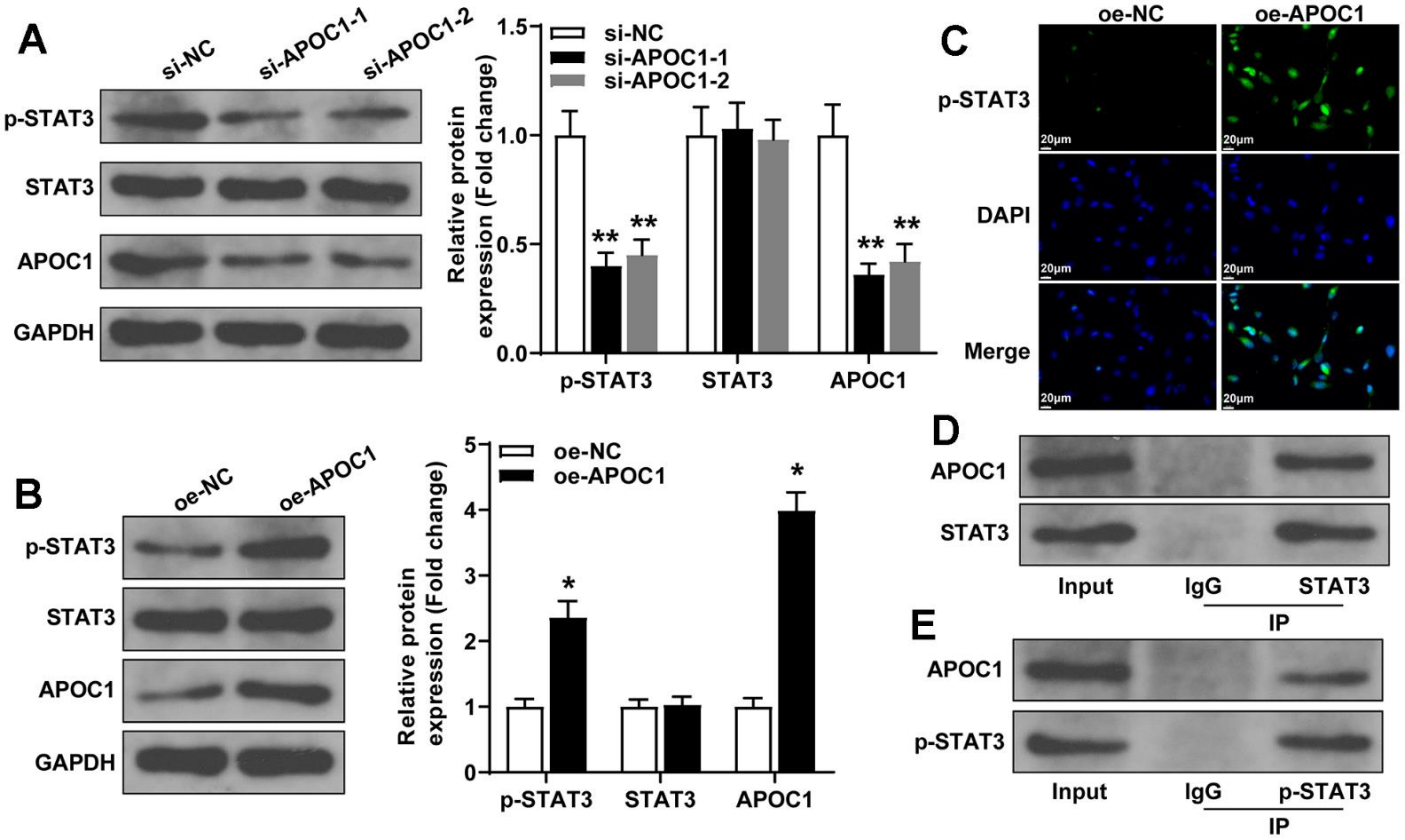
**APOC1 binds STAT3 to regulate the activation of STAT3 signal pathway.** Note: (**A**, **B**) western blot detected the expressions of APOC1, p-STAT3 and STAT3 after cell transfection with APOC1 overexpression or APOC1 knockdown, *P < 0.05, ** P < 0.01. (**C**) Immunofluorescence detected the expression of p-STAT3 in rat primary cardiomyocytes, scale bar of 25 μm; (**D**, **E**) Co-IP detected the binding of APOC1 with STAT3 and p-STAT3. Each experiment was repeated for 3 times.

APOC1 was found to activate STAT3 signal pathway in clear-cell renal cell carcinoma [18], therefore in this study, we explored the possible interaction between APOC1 and STAT3 signal pathway in cardiomyocytes. The detection by western blot showed that overexpression of APOC1 can increase the expressions of APOC1 and p-STAT3, while knockdown of APOC1 can inhibit the expressions of APOC1 and p-STAT3, but overexpression or knockdown expression of APOC1 showed little effect on the expression of STAT3 (Figure 5A, 5B). IF showed that overexpression of APOC1 can increase p-STAT3 expression and promote STAT3 nuclear translocation, different effect was found in cells transfected with APOC1 knockdown plasmid (Figure 5C). Co-IP showed that APOC1 protein can be enriched by STAT3 antibody and p-STAT3 antibody, suggesting APOC1 can directly bind STAT3 and p-STAT3 (Figure 5D, 5E). Taken together, APOC1 in cardiomyocytes can bind STAT3 to promote STAT3 phosphorylation and nuclear translocation, to further activate STAT3 signal pathway.

## DISCUSSION

Recent studies have shown the great value of STDP for the clinical treatment of stable coronary artery disease [19], PCI-related adverse cardiovascular events [20]. In this study, high-throughput sequencing was carried out to screen differentially expressed genes in the rat model of CME that are involved in the regulatory effect of STDP on CME. Accordingly, APOC1 has identified as an up-regulated gene in the CME model while being reduced by treatment with STDP. Mechanistically, APOC1 could bind to STAT3 to activate the STAT3 pathway, whereby counteracting the protective effect of STDP against CME-related myocardial injury.

Initially, our experimental data evidenced that STDP contributed to beneficial effects against myocardial damage in the rat model of CME as well as LPS-induced *in vitro* myocardial injury and inflammation, which was partially consistent with the previous findings [21, 22]. Different from our study, Zhang et al. mainly focused on peripheral microcirculation and the possible mechanism related to mitochondrial dysfunction [22]. Another study has suggested the *in vitro* cardioprotective effect of Na_2_S_2_O_4_-caused hypoxia-reoxygenation injury, with the results mainly focusing on apoptotic molecular mechanisms [21], which lacks *in vivo* validation. The study of Liu has discussed the anti-inflammatory, anti-apoptosis, and anti-oxidant mechanisms of STDP in the rat coronary microcirculatory dysfunction model; their data have demonstrated that regulation of inflammatory cytokines, apoptotic markers, and oxidative stress markers confers suppressive effect of STDP pre-treatment on inflammation, myocardial ischemic damage, and microthrombosis [23]. STDP treatment was revealed to suppress the levels of inflammatory proteins IL-6, IL-1β, and TNF-α as well as cardiac markers cTnI and CK-MB, further indicative of its anti-inflammatory and cardioprotective actions. Herein, this study mainly investigated the possible mechanism of STDP in mediating myocardial injury.

Through high-throughput sequencing, APOC1 was up-regulated in the myocardial tissues of rats rendered with CME, while STDP treatment could markedly reduce APOC1 expression. APOC1, which belongs to the apolipoprotein family, crucially participates in lipoprotein metabolism [24, 25]. ApoC1 has been reported as a potent cholesteryl ester transfer protein (CETP) inhibitor in plasma, which is highly correlated with the risk of cardiovascular disorders such as CHD since CETP can enhance the cholesterol enrichment of apoB-containing lipoproteins [26], yet its role in myocardial injury remains undefined. Since rescue experiments showed that APOC1 re-expression counteracted the anti-inflammatory and anti-apoptotic effects of STDP on myocardial damage in the rat model of CME, suggesting that STDP ameliorated LPS-elicited myocardial inflammation and apoptosis through APOC1.

At last, but not least, STAT3 pathway was proposed to be an important pathway in the treatment of STDP for CME. STAT3 pathway has been reported as a promoter of cardiomyocyte apoptosis while blocking this pathway contributes to suppression of cardiomyocyte apoptosis, thus protecting against acute myocardial infarction [27]. NDRG4 negatively mediates the activation of the JAK2/STAT3 pathway to protect H9C2 cells from myocardial infarction-caused apoptosis [28]. A recent study has identified an ApoC1-STAT3 pathway in the metastasis of clear cell renal cell carcinoma [18], yet their interaction in CME remains unknown. The current study has demonstrated that STAT3 activation in the rats with CME could be suppressed by STDP treatment, while this blockade attenuated inflammatory damage to myocardial tissues. APOC1 re-expression augmented the expression of p-STAT3, thus promoting its phosphorylation and nuclear translocation, which might be the mechanism underlying the protection against myocardial inflammation and damage induced by CME. A relation between STAT3 and LPS-evoked inflammation has been discussed in a prior study. STAT3 was proposed as a transcription factor that positively mediates the levels of pro-inflammatory cytokine IL-6; non-toxic sulfur (NTS) contributes to protection against inflammation through inhibition of LPS-induced TLR-4, JAK2, STAT3, and IL-6 [29].

All in all, the current study highlights a new cardioprotective mechanism of STDP in the setting of CME, in which STDP downregulates the level of APOC1 and impairs the APOC1-dependent STAT3 pathway activation. Furthermore, we integrated high-throughput sequencing results with the results of animal experiments for the identification of molecular targets involved in the TCM treatment of CME, which may give insight into the development of new protective targets. The cardioprotective mechanism of STDP in CME through APOC1-dependent STAT3 pathway was verified only in cardiomyocytes injury models and *in vivo* experiment validation on this mechanism will be one of our future directions.
